# NFATc1 Regulation of TRAIL Expression in Human Intestinal Cells

**DOI:** 10.1371/journal.pone.0019882

**Published:** 2011-05-16

**Authors:** Qingding Wang, Yuning Zhou, Heidi L. Weiss, Chi-Wing Chow, B. Mark Evers

**Affiliations:** 1 Department of Surgery, The University of Kentucky, Lexington, Kentucky, United States of America; 2 Markey Cancer Center, The University of Kentucky, Lexington, Kentucky, United States of America; 3 Department of Molecular Pharmacology, Albert Einstein College of Medicine, Bronx, New York, United States of America; Virginia Commonwealth University, United States of America

## Abstract

TNF-related apoptosis-inducing ligand (TRAIL; Apo2) has been shown to promote intestinal cell differentiation. Nuclear factor of activated T cells (NFAT) participates in the regulation of a variety of cellular processes, including differentiation. Here, we examined the role of NFAT in the regulation of TRAIL in human intestinal cells. Treatment with a combination of phorbol 12-myristate 13-acetate (PMA) plus the calcium ionophore A23187 (Io) increased NFAT activation and TRAIL expression; pretreatment with the calcineurin inhibitor cyclosporine A (CsA), an antagonist of NFAT signaling, diminished NFAT activation and TRAIL induction. In addition, knockdown of NFATc1, NFATc2, NFATc3, and NFATc4 blocked PMA/Io increased TRAIL protein expression. Expression of NFATc1 activated TRAIL promoter activity and increased TRAIL mRNA and protein expression. Deletion of NFAT binding sites from the TRAIL promoter did not significantly abrogate NFATc1-increased TRAIL promoter activity, suggesting an indirect regulation of TRAIL expression by NFAT activation. Knockdown of NFATc1 increased Sp1 transcription factor binding to the TRAIL promoter and, importantly, inhibition of Sp1, by chemical inhibition or RNA interference, increased TRAIL expression. These studies identify a novel mechanism for TRAIL regulation by which activation of NFATc1 increases TRAIL expression through negative regulation of Sp1 binding to the TRAIL promoter.

## Introduction

The mammalian intestinal mucosa undergoes a process of continual renewal, characterized by active proliferation of stem cells localized near the base of the crypts, progression of these cells up the crypt-villus axis with cessation of proliferation and subsequent differentiation into one of the four primary cell types [1]. In the process of differentiation, enterocytes acquire structural features of mature cells, such as microvilli, and express specific gene products such as brush border enzymes [2]. The differentiated enterocytes, which make up the majority of the cells of the gut mucosa, then undergo a process of programmed cell death (i.e., apoptosis) and are extruded into the gut lumen [3]. The cellular mechanisms regulating this tightly regimented process have not been clearly defined.

Tumor necrosis factor-related apoptosis-inducing ligand (TRAIL; also called Apo-2 ligand), a novel member of the TNF family, was identified based on homology to the extracellular domains of TNF and FasL (CD95L) [4], [5]. Unlike TNF and FasL, TRAIL is expressed in a variety of cell types and is capable of inducing apoptosis in neoplastic cells [6]. In addition, TRAIL blockade results in hyperproliferation of synovial cells and lymphocytes, whereas TRAIL inhibits DNA synthesis in lymphocytes by blocking cell cycle progression [7]. Recently, TRAIL has been shown to promote dendritic cell differentiation [8]. We have found that inhibition of phosphatidylinositol 3-kinase (PI3-kinase) or overexpression of PTEN enhances intestinal cell differentiation [9] and increases TRAIL expression in intestinal cells [10]. Moreover, TRAIL is expressed in the differentiated region of the small bowel and colonic mucosa. Exposure of the fetal human intestine cell line, tsFHI, to recombinant TRAIL increased the expression levels of the canonical differentiation marker dipeptidylpeptidase IV (DPPIV) and the cyclin-dependent kinase inhibitors p21^Waf1^ and p27^Kip1^ [11], which mediate the induction of growth arrest and the stabilization of differentiated traits, respectively. Together, these studies demonstrate an important role for TRAIL in the regulation of intestinal cell differentiation.

The nuclear factor of activated T cells (NFATc) proteins are a family of transcription factors whose activation is controlled by calcineurin, a calcium-dependent phosphatase. Four distinct genes encoding closely related NFATc proteins (NFATc1–4) [12] have been identified and are involved in multiple biological processes ranging from lymphocyte activation and development to cardiac hypertrophy [13]. NFAT, which exists in a highly phosphorylated form in the cytoplasm, translocates into the nucleus upon dephosphorylation by the phosphatase calcineurin in response to increases in intracellular calcium, where it binds to enhancer elements of specific genes leading to transcriptional activation [14]. Calcineurin activity can be blocked by cyclosporin A (CsA), thereby preventing the nuclear translocation of NFAT. NFAT plays a critical role in the regulation of T cell receptor-mediated CD95 ligand expression [15] and has been shown to regulate cell differentiation and development in a number of cell types. For example, NFAT regulates the development of the cardiovascular system [16]. Primary keratinocyte cell differentiation is associated with nuclear localization of NFAT; this effect is blocked by CsA [17]. NFAT also plays a role in adipocyte differentiation [18] and stimulation of myogenic differentiation via activation of calcineurin [19]. Recently, we have shown that activation of NFAT increases PTEN and p27^kip1^ expression and decreases Akt phosphorylation and that NFAT activation is required for sodium butyrate mediated-intestinal cell differentiation [20]. However, the NFAT target genes, which may contribute to intestinal differentiation, are not known.

Previously, we cloned the human TRAIL promoter and identified a number of putative transcription factor binding sites including NFAT and Sp1 sequences [21]. Given the important role of NFAT in tissue differentiation and the fact that NFAT binding sites are located in the TRAIL promoter, we determined whether NFAT plays a role in TRAIL regulation. Here, we show induction of TRAIL expression in intestinal-derived cells by activation of the NFAT pathway. Inhibition of NFAT decreased TRAIL protein expression while overexpression of NFATc1 increased TRAIL promoter activity and increased TRAIL protein and mRNA expression. Deletion of putative NFAT binding sites in the TRAIL promoter did not affect NFATc1 induction of TRAIL promoter activity. Knockdown of NFATc1 increased Sp1 transcription factor binding and, importantly, inhibition or knockdown of Sp1 increased TRAIL expression. These findings demonstrate that activation of NFAT increases TRAIL expression through negative regulation of Sp1 binding to the TRAIL promoter in human intestinal cells.

## Results

### NFAT activation increases TRAIL expression in HT29 cells

**Figure 1:**
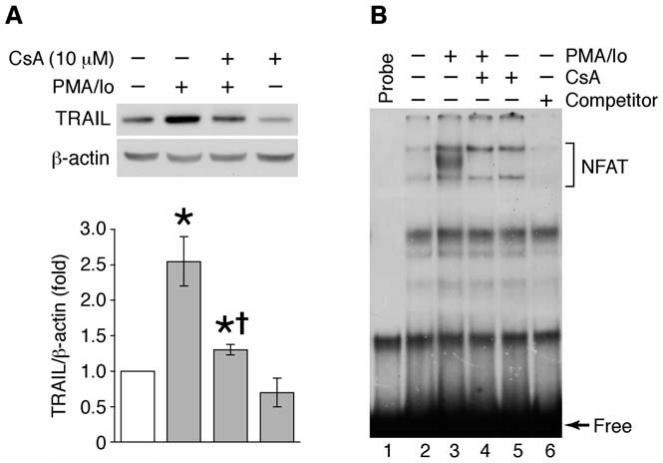
PMA/Io-mediated induction of TRAIL expression was attenuated by cyclosporin A, a potent calcineurin inhibitor in HT29 cells. (**A**) HT29 cells were pre-treated with cyclosporin A (CsA) for 30 min followed by the combination of PMA (100 nM) plus Io (2.5 µM) with CsA for 2 h. Total protein was extracted from cells and resolved by SDS-PAGE, transferred to a PVDF membrane and probed with anti-TRAIL and anti-β-actin antibodies. TRAIL signals from three separate experiments were quantitated densitometrically and expressed as fold-change with respect to β-actin. (Data shown as mean ± standard error of the mean; *, *P*<0.05, PMA/Io or PMA/Io plus CSA vs. control; ^+^, *P*<0.05, PMA/Io plus CSA vs. PMA/Io). (**B**) Cells were treated with PMA/Io for 60 min in the presence or absence of CsA. Nuclear protein was extracted and NFAT DNA binding was analyzed by EMSA. Unlabeled NFAT oligonucleotide was added in molar excess to confirm binding specificity (*competitor*).

Previously, we have shown that inhibition of PI3-kinase, which augments enterocyte-like differentiation of HT29 and Caco-2 human colon cancer cells [9], increased TRAIL expression [10]. The importance of TRAIL in intestinal cell differentiation has subsequently been demonstrated [11]. In our current study, we have investigated the cellular mechanisms regulating TRAIL expression in these intestinal-derived cell lines. HT29 cells were pretreated with CsA, an inhibitor of calcineurin [22], followed by treatment with PMA (100 nM) plus Io (2.5 µM), pharmacological agents that activate NFAT in intestinal cell types [23], in the presence or absence of CsA for 2 h. Whole cell lysates were analyzed by Western blot using anti-TRAIL antibody (Figure 1A). PMA/Io treatment resulted in the induction of TRAIL expression compared with control cells treated with vehicle (i.e., Me_2_SO); this induction was attenuated by pretreatment with CsA. To test whether PMA/Io activates NFAT, we performed EMSAs with nuclear extracts obtained from HT29 cells treated for 60 min with PMA/Io using a commercial oligonucleotide with consensus binding sites for NFAT as the probe. As shown in Figure 1B, treatment with PMA/Io increased NFAT binding activity. As expected, the formation of the increased binding complex was abolished in extracts from cells pretreated with CsA. The specificity of the complex was determined using unlabeled cold probe as a competitor. Our results suggest a role for NFAT activation in TRAIL induction in intestinal cells.

### Knockdown of NFAT blocks PMA/Io induced TRAIL expression

**Figure 2:**
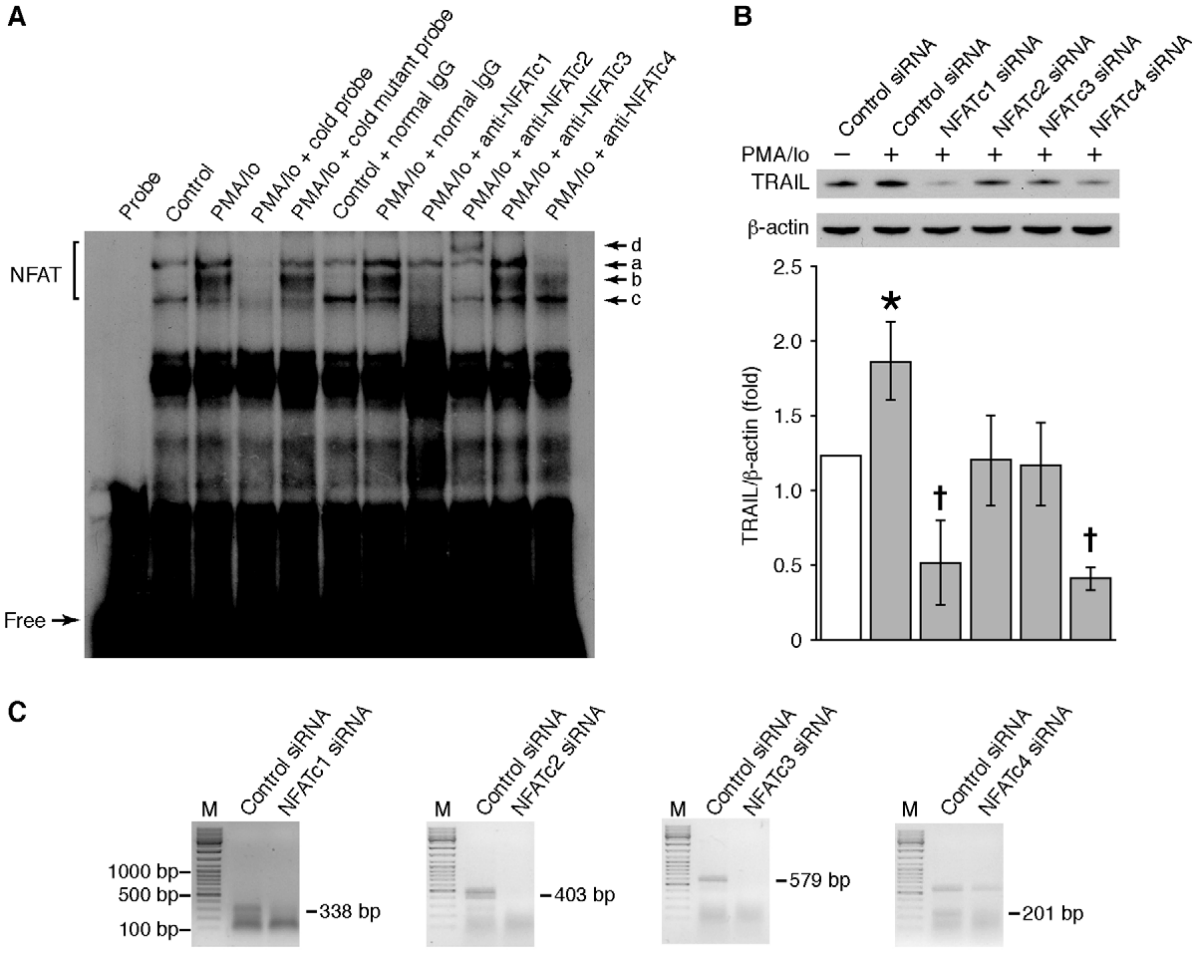
Knockdown of NFAT blocked PMA/Io-induced TRAIL expression in HT29 cells. (**A**) HT29 cells were treated with PMA/Io for 60 min and NFAT DNA binding activity was assessed by EMSA using nuclear extracts. In addition, nuclear extracts were incubated with ^32^P-labeled NFAT specific DNA probe alone or in the presence of unlabeled wild type (*cold*) NFAT oligonucleotide or mutant NFAT oligonucleotide or specific antibodies to either NFATc1, NFATc2, NFATc3, or NFATc4. (**B**) HT29 cells were transfected with NFATc1, c2, c3, c4 or control siRNA. After a 48 h incubation, transfected cells were treated with PMA/Io for 2 h. Total protein was extracted and TRAIL expression levels were determined by Western blotting using anti-TRAIL antibody. The membrane was stripped and reprobed using anti-β-actin antibody to confirm equal loading. TRAIL signals from three separate experiments were quantitated densitometrically and expressed as fold-change with respect to β-actin. (Data shown as mean ± standard error of the mean; *, *P*<0.05, PMA/Io plus control siRNA vs. control siRNA; ^+^, *P*<0.05, PMA/Io plus NFATC1 siRNA or NFATC4 siRNA vs. PMA/Io plus control siRNA). (**C**) To confirm NFAT suppression, total RNA was extracted from cells and NFAT expression was assessed by RT-PCR.

Four classical isoforms of NFAT have been identified [12]. To determine which of the NFAT isoforms are involved in TRAIL regulation, EMSAs were performed. As shown in Figure 2A, treatment with PMA/Io increased NFAT binding activity as noted by increased density of band *b*. The specificity of this binding was confirmed by competition studies using cold wild type and mutant competitor probes. Addition of anti-NFATc1 antibody to the mixture abolished bands *b* and *c*. Band *b* was also abolished when anti-NFATc2 antibody was added to the mixture and a supershifted band (band *d*) was observed. When anti-NFATc4 antibody was added to the mixture, band *a* was abolished; there was no effect on the binding complex with addition of anti-NFATc3 antibody. These results demonstrate that NFATc1, NFATc2, and NFATc4 isoforms are present in HT29 cells. Treatment with PMA/Io increased band *b*, suggesting a role of NFATc1 or NFATc2 in PMA/Io induction of TRAIL expression in HT29 cells.

To confirm the contribution of each isoform to TRAIL expression, individual NFAT isoforms were silenced by transfection of HT29 cells with the relevant siRNA. As shown in Figure 2B, transfection of NFATc2 or NFATc3 siRNA blocked PMA/Io increased TRAIL protein expression, while transfection of NFATc1 or NFATc4 siRNA not only blocked PMA/Io induced TRAIL expression but also decreased basal TRAIL protein expression compared with cells transfected with non-targeting control siRNA. The knockdown of individual NFAT isoforms was confirmed by RT-PCR as shown in Figure 2C. The results indicate that, although NFATc1 and NFATc2 are important for PMA/Io increased TRAIL expression, NFATc3 and NFATc4 may also play a role in TRAIL regulation in human intestinal cells.

### Overexpression of NFATc1 increases TRAIL expression in HT29 and Caco-2 cells

**Figure 3:**
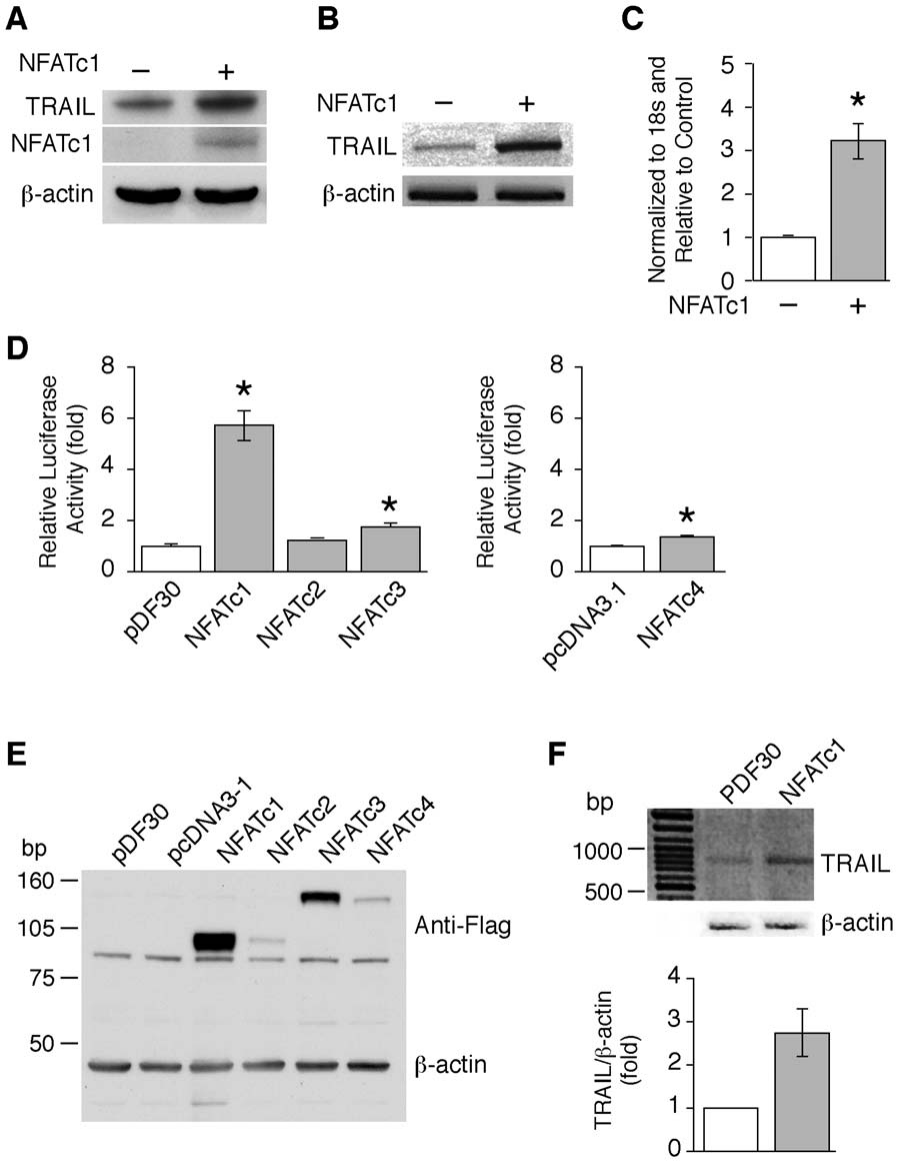
Overexpression of NFATc1 increased TRAIL expression in HT29 and Caco-2 cells. HT29 cells were transfected with either the control plasmid (pDF30) or Flag-tagged NFATc1. At 48 h posttransfection, cells were harvested. (**A**) Expression of TRAIL and NFATc1 protein was detected by Western blot using anti-TRAIL or anti-Flag antibodies. Membranes were reprobed with anti-β-actin antibody to assess loading. (**B and C**) Total RNA was extracted, and RT-PCR (**B**) or real time PCR (**C**) performed. (Data shown as mean ± standard error of the mean. *, *P*<0.05, vs. control plasmids). (**D**) Caco-2 cells were transfected with a proximal 1371 bp TRAIL promoter construct together with either control plasmid pDF30, pCDNA3.1 or Flag-tagged NFATc1, NFATc2, NFATc3 or NFATc4. At 48 h posttransfection, cells were harvested; luciferase activity was then assayed. All results were normalized for transfection efficiency using the pRL-Tk-luc plasmid (Promega). (Data shown as mean ± standard error of the mean. *, *P*<0.05, vs. control plasmids). (**E**) Expression of NFATc1, NFATc2, NFATc3 and NFATc4 in Caco-2 cells was confirmed by Western blot using anti-Flag antibody. (**F**) Caco-2 cells were transfected with control plasmid, pDF30, or Flag-tagged NFATc1. At 48 h posttransfection, total RNA was extracted, and RT-PCR performed using primers to human TRAIL and β-actin. TRAIL signals from three separate experiments were quantitated densitometrically and expressed as fold-change with respect to β-actin (Data shown as mean ± standard error of the mean).

To further determine the role of NFAT in TRAIL regulation, HT29 cells were transfected with a plasmid encoding NFATc1, or the control pDF30 vector; TRAIL protein expression was assayed by Western blotting. Overexpression of NFATc1 by transfection with the pSH-NFATc1 vector increased TRAIL protein expression compared with the control pDF30 vector (Figure 3A). NFATc1 overexpression was confirmed using anti-NFATc1 antibody. To address whether TRAIL mRNA induction paralleled the increase in TRAIL protein, RT-PCR (Figure 3B) and real time PCR (Figure 3C) were performed with total RNA extracted from transfected HT29 cells; TRAIL mRNA induction was noted with NFATc1 overexpression.

To determine whether NFAT induction of TRAIL expression was regulated at the level of promoter activity, Caco-2 human colon cancer cells were transfected with plasmids encoding NFATc1, NFATc2, NFATc3, NFATc4 or control vectors pDF30 or pcDNA3.1 together with a 1371 bp TRAIL proximal promoter construct [21]. Overexpression of NFATc1 resulted in an induction of TRAIL promoter activity, whereas NFATc3 and NFATc4 overexpression only slightly increased TRAIL promoter activity (Figure 3D). The overexpression of Flag-tagged NFAT protein expression was confirmed by Western blotting using anti-Flag antibody (Figure 3E). RT-PCR was performed with total RNA extracted from NFATc1 transfected Caco-2 cells; similar to HT29 cells, overexpression of NFATc1 increased TRAIL mRNA levels in Caco-2 cells (Figure 3F). Taken together, these results demonstrate both TRAIL mRNA and protein induction by the NFAT signaling pathway in HT29 and Caco-2 human colon cancer cells.

### Identification and functional assessment of putative NFAT binding sites

**Figure 4:**
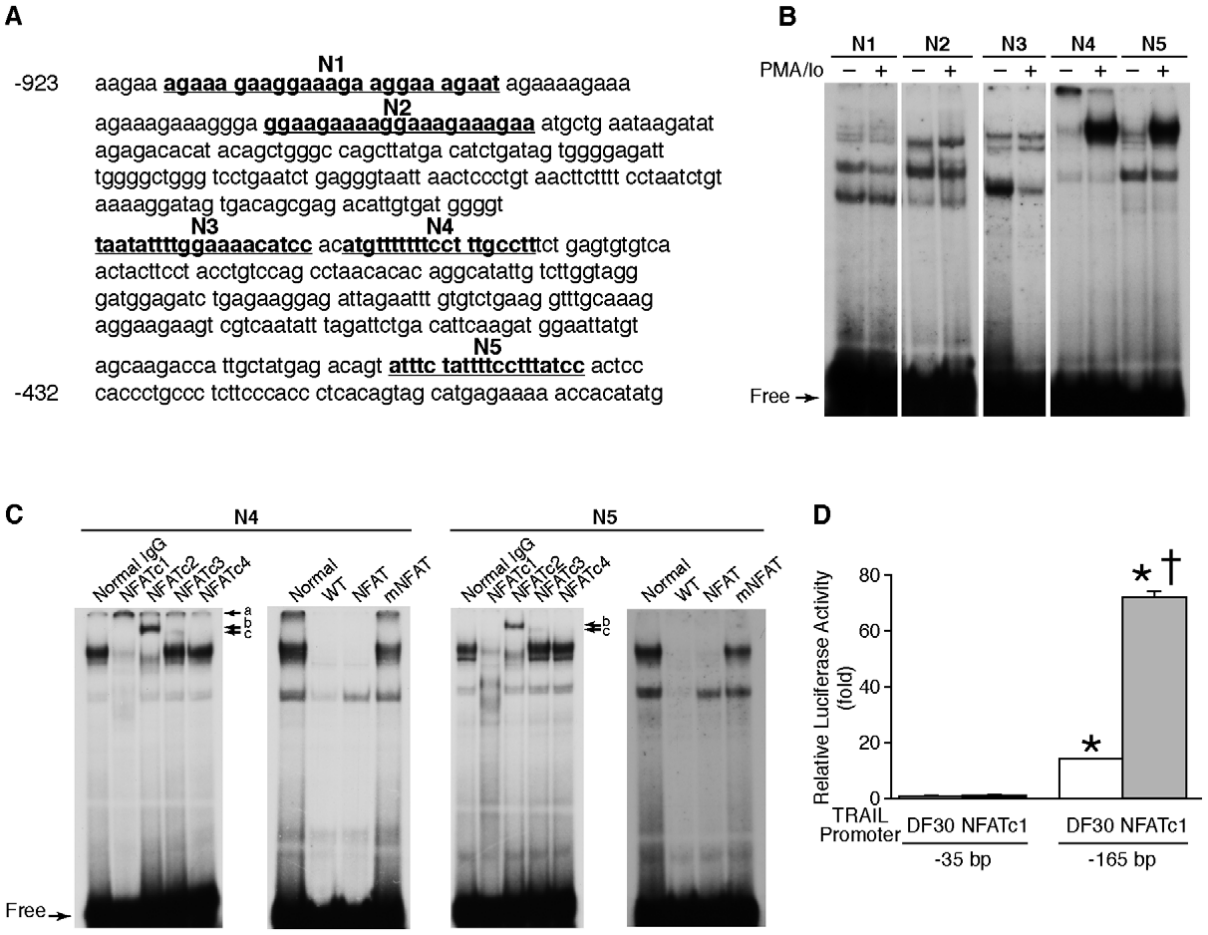
Deletion of NFAT binding sites did not eliminate NFATc1 increased TRAIL promoter activity. (**A**) Nucleotide sequences of the putative NFAT binding sites in the human TRAIL promotor. The 1371 bp TRAIL promoter contains five putative NFAT binding sites (N1, N2, N3, N4, and N5). The numbers on the left are the nucleotide positions relative to the transcriptional start site. (**B**) HT29 cells were treated with or without PMA/Io for 60 min and nuclear protein extracted; EMSA was performed with ^32^P-labeled probes spanning each of the five NFAT binding sites (N1, N2, N3, N4, and N5) in the human TRAIL promoter. (**C**) Specific binding of various NFAT isoforms with probe N4 or N5 was determined by addition of antibodies to either NFATc1, NFATc2, NFATc3, or NFATc4. Specific binding of NFAT was also confirmed by cold competition using unlabeled wild type (WT) and mutant probes at 100-fold molar excess. (**D**) Caco-2 cells were transfected with constructs containing −35 or −165 TRAIL promoter sequences together with either control plasmid pDF30, or Flag-tagged NFATc1, respectively. At 48 h posttransfection, cells were harvested; and luciferase activity was assayed. All results were normalized for transfection efficiency using the pRL-Tk-luc plasmid (Promega). (Data shown as mean ± standard error of the mean; *, *P*<0.05, DF30 or NFATc1 plus −165 bp promoter vs. DF30 or NFATc1 plus −35 promoter, respectively; ^+^, *P*<0.05, DF30 plus −165 bp promoter vs. NFATc1 plus −165 bp promoter).

Previously, we have reported two NFAT binding sites identified in the 1371 bp TRAIL promoter by computer sequence analysis using MatInspector V2.2 software [21] as shown in Figure 4A (N1 and N2). In addition, we also found three other potential NFAT binding sites, N3, N4, and N5, by computer sequence analysis using Genomatrix software. All of these five sites were located in the −923 to −383 region of the TRAIL promoter.

To determine whether PMA/Io treatment affects the binding activity of these potential NFAT binding sites, EMSA was performed. Treatment with PMA/Io increased N4 and N5, but not N1, N2, and N3 binding activity (Figure 4B), suggesting N4 and N5 may be important for TRAIL regulation. To demonstrate whether NFAT directly binds to the putative N4 or N5 NFAT binding sites, supershift assays were performed. As shown in Figure 4C, the addition of antibodies against NFATc1, NFATc2, or NFATc3 resulted in shifted bands (*a*, *b*, *c*, respectively) or diminished binding of the protein-DNA binding complex. We did not identify NFATc4 binding to these two sites.

To determine the contribution of these NFAT binding sites to TRAIL promoter activation, deletional analyses were performed. Caco-2 cells were transfected with two 5′- deletion promoter constructs together with control vector pDF30 or a plasmid encoding NFATc1. As shown in Figure 4D, deletion of NFAT binding sites from −1371 to −165 did not abrogate NFATc1 induction of the TRAIL promoter activity. Deletion from −1371 to −35 resulted in a marked decrease in basal promoter activity and eliminated NFATc1 induction. Although NFAT transcription factors bind to the TRAIL promoter, our results showed that NFATc1 increased TRAIL promoter activity independent of direct binding to the promoter. Instead, the region between −165 and −35 contains elements important for NFATc1 induction of TRAIL expression.

**Figure 5:**
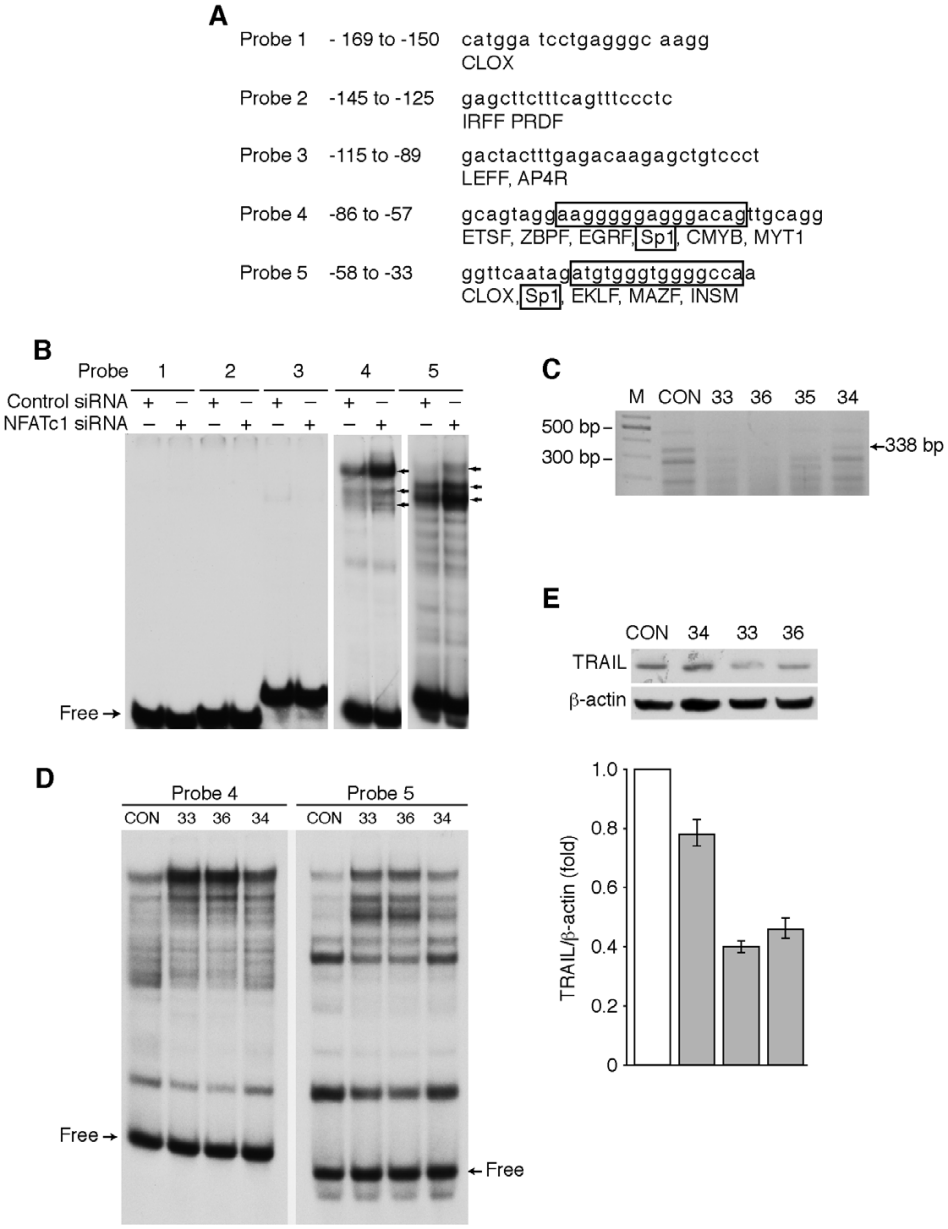
Identification of NFATc1 responsive sequences in the TRAIL promoter. (**A**) Double-stranded oligonucleotides corresponding to all of the potential transcriptional binding sites in human TRAIL promoter −169 to −33 were synthesized and used for DNA binding analysis. The putative Sp1 binding sequences are boxed, which are also overlapped with other transcription factor binding sequences. (**B**) HT29 cells were treated with or without PMA/Io for 60 min and nuclear protein extracted. Double-stranded oligonucleotides (as shown in A) were radiolabeled and tested for DNA binding by EMSA. The arrows indicated the increased binding complexes. (**C**) Total RNA from HT29 cells stably transfected with control (CON) or four individual shRNAs targeting human NFATc1 (clones 33, 34, 35, 36) was extracted and RT-PCR performed for NFATc1 mRNA expression. (**D and E**) Nuclear protein (D) or total protein (E) from HT29 cells stably transfected with control (CON) or NFATc1 shRNA (clones 33, 34, 36) was extracted. EMSA was performed (D) using probe 4 and probe 5 (sequences shown in Figure 6). Western blotting was performed for the analysis of TRAIL expression (E); membranes were stripped and re-probed with anti-β-actin to confirm equal loading. TRAIL signals from two separate experiments were quantitated densitometrically and expressed as fold-change with respect to β-actin (Data shown as mean ± standard error of the mean).

To further identify the NFATc1 responsive elements in the −165 TRAIL promoter, five oligonucleotide probes were synthesized. These probes contained all of the putative transcriptional binding sites identified between −169 and −33 by computer sequence analysis (Figure 5A). HT29 cells were transfected with non-targeting control siRNA or siRNA targeting NFATc1. Forty-eight h after transfection, cells were harvested and nuclear protein extracted and EMSAs performed. There was no obvious binding complex found using probes 1, 2 or 4. Interestingly, knockdown of NFATc1 increased the binding activities of probes 4 and 5 (Figure 5B).

To further confirm NFATc1 regulation of TRAIL expression, HT29 cells stably transfected with the shRNA targeting NFATc1 and the control shRNA (CON) were established. Complete knockdown of NFATc1 in clones 33, 36 and 35 and partial knockdown of NFATc1 in clone 34 were confirmed in HT29 cells transfected with NFATc1 shRNA by RT-PCR (Figure 5C). The increase of probe 4 and probe 5 binding activities by blockade of NFATc1 was demonstrated by EMSA (Figure 5D). Consistently, decreased TRAIL protein expression levels were noted in these stable cell lines transfected with NFATc1 shRNA, compared with cells transfected with control shRNA detected by Western blotting (Figure 5E). These results suggested that probes 4 and 5 contain elements important for NFATc1 induction of TRAIL expression.

### NFATc1 regulates TRAIL expression through negative regulation of Sp1 binding to the TRAIL promoter

**Figure 6:**
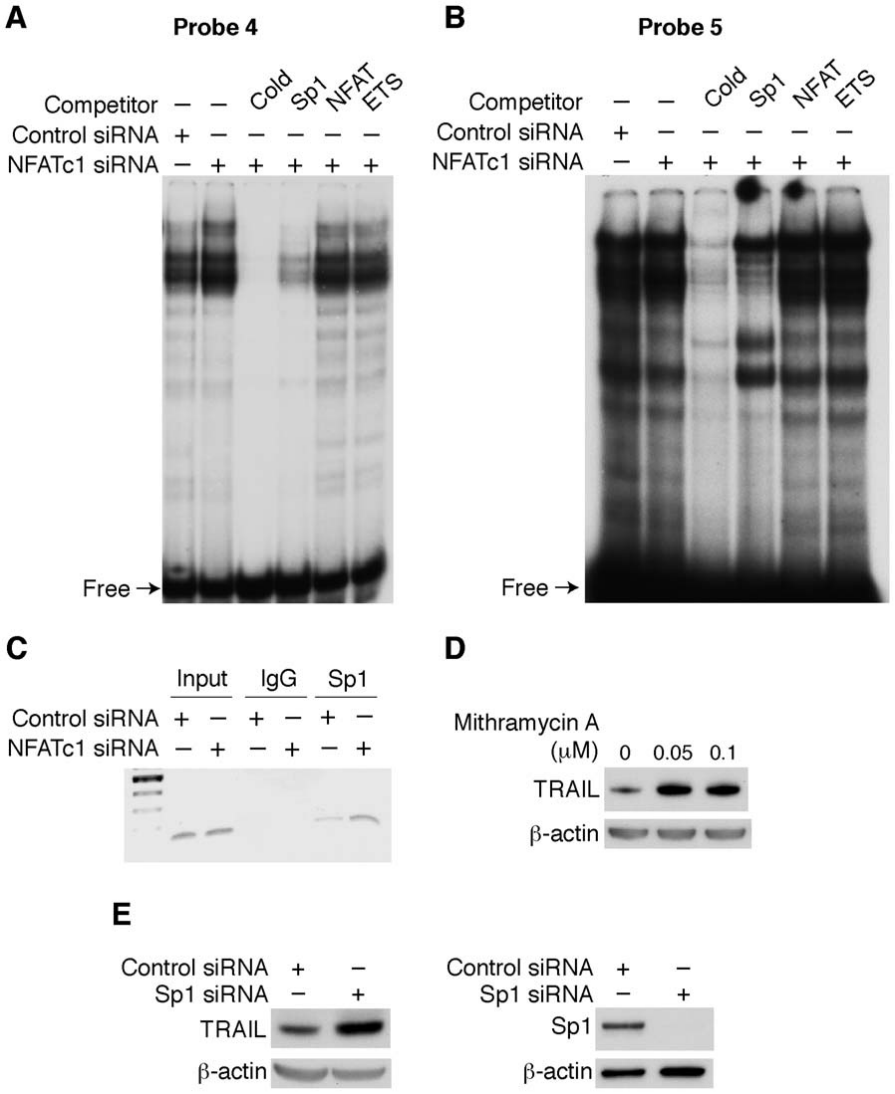
Sp1 mediated NFATc1 regulation of TRAIL expression. HT29 cells were transiently transfected with control siRNA or siRNA targeting NFATc1. Nuclear protein was extracted and EMSAs performed. Nuclear extracts were incubated with ^32^P-labeled probe 4 (**A**) or probe 5 (**B**) alone or in the presence of unlabeled wild type (*cold*) probe 4 or probe 5, the Sp1 oligonucleotide, the NFAT oligonucleotide, or the ETS oligonucleotide, respectively. (**C**) HT29 cells were subjected to ChIP assay; soluble chromatin was prepared from HT29 cells transfected with control siRNA or siRNA targeting NFATc1 and immunoprecipitated with Sp1 antibody or IgG. Total (Input) and immunoprecipitated DNAs were then PCR-amplified using primer pairs covering the Sp1 binding sites within the human TRAIL promoter. (**D**) HT29 cells were treated with the Sp1 inhibitor mithramycin for 24 h and total protein extracted and Western blotting performed using anti-TRAIL antibody. (**E**) HT29 cells were transiently transfected with control siRNA or siRNA targeting Sp1. Forty-eight h after transfection, total protein was extracted and Western blotting performed to assess TRAIL expression (left panel) or to confirm knockdown of Sp1 (right panel). Membranes were stripped and re-probed with anti-β-actin antibody to confirm equal loading.

Since both probe 4 and probe 5 contain putative Sp1 binding sites (Figure 5A), we next determined whether Sp1 protein comprised part of the increased binding complex to probes 4 and 5 associated with NFATc1 knockdown. Nuclear extracts from HT29 cells transfected with non-targeting control siRNA or siRNA targeting NFATc1 were isolated and EMSAs performed. As shown in Figure 6, A and B, the specificity of the complex was determined using unlabeled oligonucleotide as a competitor. In addition, when an unlabeled, commercially available Sp1 probe was added to the mixture, the increased binding complex was significantly diminished. In contrast, addition of unlabeled NFAT or ETS probes did not alter the binding complex. NFATc1 knockdown did not affect the Sp1 protein expression level (Wang et al., data not shown); therefore, increased Sp1 binding to the TRAIL promoter may not be through increased Sp1 protein expression.

To further confirm these findings, occupancy of the TRAIL promoter by Sp1 in cells was further analyzed using a ChIP assay. Cross-linked chromatin was prepared from HT29 cells transfected with control siRNA or siRNA targeting NFATc1. The human TRAIL promoter region containing the Sp1 binding sites was precipitated using either the anti-Sp1 antibody or IgG, and using the probe 1 (forward) and the probe 5 (reverse) shown in Figure 5A as gene-specific primers and the sequence (136 bp) encompassing the Sp1 binding sites was amplified. As shown in Figure 6C, the binding of the TRAIL promoter and Sp1 was confirmed and consistent with our EMSA results, knockdown of NFATc1 increased Sp1 binding to TRAIL promoter. These results demonstrate that knockdown of NFATc1 increased Sp1 protein binding to the −165/−35 region of the TRAIL promoter.

Since Sp1 protein directly binds to the TRAIL promoter and knockdown of NFATc1 decreased TRAIL expression and increased Sp1 binding, we next investigated whether Sp1 transcription factor regulates TRAIL expression. Mithramycin has affinity for the GGGCGG binding sequence, and effectively blocks the binding of Sp1 [24]. HT29 cells were treated with mithramycin for 24 h and total protein extracted; TRAIL expression was determined by Western blotting. As shown in Figure 6D, treatment with mithramycin significantly increased TRAIL expression in HT29 cells. Sp1 binding to the TRAIL promoter has been demonstrated [25]. To further delineate Sp1 transcription factor regulation of TRAIL expression, HT29 cells were transfected with non-targeting control siRNA or siRNA targeting Sp1. Forty-eight h after transfection, cells were harvested and total protein extracted and Western blotting performed. In agreement with the Sp1 inhibition, transfection of Sp1 siRNA (Figure 6E) significantly increased TRAIL protein expression. Knockdown of Sp1 was confirmed by Western blotting using anti-Sp1 antibody. Collectively, these results demonstrate that NFATc1 regulated Sp1 protein binding to the TRAIL promoter and thus, regulated TRAIL expression.

## Discussion

Previously, we demonstrated that intestinal cell differentiation is associated with the induction of TRAIL expression [10]. Subsequently, Rimondi et al. [11] showed that TRAIL promotes intestinal cell differentiation. Analysis of the TRAIL promoter identified several putative transcription factor binding sites, including NFAT, which might be involved in the regulation of TRAIL expression [21]. Here, we show that activation of the NFATc1 isoform increased TRAIL expression, not through the direct binding to the TRAIL promoter, but indirectly through the regulation of Sp1 binding.

Although five NFAT transcription factor binding sites were identified in the TRAIL promoter, our results showed that NFATc1 increased TRAIL promoter activity not by direct binding but, alternatively, through negative regulation of Sp1 binding to the promoter. Recently, we have found that knockdown of NFATc1 decreased PTEN expression and increased the phosphorylation levels of Akt while overexpression of NFATc1 increased PTEN expression and decreased Akt phosphorylation in HT29 and Caco-2 cells [20]. In addition, our previous findings demonstrated that inhibition of PI3-kinase or overexpression of PTEN enhanced intestinal cell differentiation [9] and increased TRAIL expression in intestinal cells [10]. These results suggest that NFATc1 regulates TRAIL expression through the induction of PTEN induction. Activation of PI3-kinase increases Sp1 activation in various cell types [26], [27], [28], [29]. We also have found that overexpression of PTEN inhibits Sp1 reporter activity in HT29 and Caco-2 cells (unpublished data). Moreover, PTEN downregulates p75NTR expression by decreasing DNA-binding activity of Sp1 [30]. PTEN dephosphorylates the Sp1 transcription factor [31], the phosphorylation status of which directly impacts its ability to bind to some DNA promoter regions [32], [33]. Therefore, it is reasonable to postulate that activation of NFATc1 increases PTEN expression and, as a result, decreases Sp1 binding to TRAIL promoter and increases TRAIL expression. Although knockdown of NFATc1 increased Sp1 binding to the TRAIL promoter, knockdown of NFATc1 did not affect Sp1 protein expression levels, suggesting that the increased Sp1 protein binding to the TRAIL promoter, associated with NFATc1 knockdown, may not be through increased Sp1 protein expression.

Similar to our findings showing induction of TRAIL by NFAT, activation of NFAT isoforms, including NFATc1, up-regulated FasL expression [34], [35] and TNFα expression [36] in several cell types. NFAT has been shown to play a pro-apoptotic role through the up-regulation of FasL [35], [37]. Taken together with our current findings, these studies demonstrate that the activation of NFAT results in the induction of apoptotic-related proteins in various cell types. Recently, activation of NFATc1 was noted specifically in the mononuclear cells of the inflamed colonic mucosa from patients with ulcerative colitis [38]. Moreover, increased expression of TRAIL and FasL has been identified in the intestinal epithelial cells and colonic lamina propria, respectively, associated with inflammatory bowel disease (IBD) [39], [40]. It is well known that TNF is one of the most potent effector cytokines in the pathogenesis of IBD [41]. It is possible that induction of these TNF family members is due, in part, to the activation of NFATc1 in the abnormal intestinal cells in patients with IBD. Given the fact that CsA is currently an effective therapy for patients with IBD [42], NFAT-dependent induction of TNF family members may play an important role in the progression of this disease.

Sp1 recognizes and binds to GC/GT boxes and regulates a large number of mammalian genes in normal and transformed cells [43]. Activation of Sp1 not only increases but also represses expression of certain genes. For example, Sp1 has been shown to bind to the promoter and repress transcription of the human telomerase reverse transcriptase [44] and the human β-like globin gene expression [45]. In addition, Sp1 can repress the expression of certain genes by recruiting histone deacetylase 1 (HDAC1) to deacetylate histones [44], [46]. Consistent with these findings, our results show that Sp1 binds to the TRAIL promoter and that inhibition of Sp1 by mithramycin or siRNA targeting Sp1 significantly increased TRAIL expression, suggesting a negative regulation of TRAIL expression by Sp1 in human intestinal cells. It is likely that inhibition or knockdown of Sp1 may diminish the promoter repression and result in TRAIL induction.

Sp1 binds the Myc-associated zinc finger protein (MAZ) promoter region and represses promoter activity [47]. It has been reported that methylation plays an important role in this suppression of transcription and that the interaction of Sp1 with DNA methyltransferase (DNMT) contributes to Sp1-mediated repression. DNMT inhibitors induce TRAIL expression in various cell types [25], [48]. Moreover, inhibition of DNMT restores PTEN expression by epigenetic mechanisms and results in the inhibition of the PI3-kinase/Akt pathway [49], an important signaling pathway that we and others have shown regulates TRAIL expression [10], [48]. Whether DNMT is involved in Sp1 regulation of TRAIL expression and the cross-talk between Sp1 and PTEN/PI3-kinase/Akt signaling deserve to be further investigated. In contrast to our findings, Sp1 has been shown to mediate HDAC inhibitor (HDACi) induction of TRAIL expression. Mutation of Sp1 binding sites blocked HDACi-induced TRAIL promoter activity [25], [50] and protein expression [50] in acute myeloid leukemia and human breast cancer cells. However, knockdown of Sp1 did not decrease basal TRAIL protein expression [50], suggesting a differential regulation of basal and HDACi-induced TRAIL expression in these cell lines.

In summary, our current study identifies the NFAT molecular pathway as an important regulator of TRAIL. Furthermore, we demonstrate that Sp1 inhibition increases TRAIL expression. Importantly, the induction of TRAIL expression by NFATc1 is not through the direct binding of NFATc1 to the TRAIL promoter but through negative regulation of Sp1 binding. TRAIL has been shown to play an important role in intestinal cell differentiation [11]. Together with our previous findings showing the importance of NFATc1 in the regulation of PTEN expression and intestinal cell differentiation [10], our current study suggests that TRAIL, as a downstream target of NFAT signaling, may be involved in NFAT-mediated regulation of intestinal cell differentiation.

## Materials and Methods

### Materials

Phorbol 12-myristate 13-acetate (PMA) was purchased from Sigma Chemical Company (St. Louis, MO). A23187 calcium ionophore (Io) was from Alexis Corporation (San Diego, CA). The calcineurin inhibitor cyclosporine A (CsA) was from Calbiochem (San Diego, CA). The plasmids encoding Flag-tagged NFATc1, NFATc2, NFATc3 and empty vector (pDF30) were kindly provided by Dr. Gerald R. Crabtree (Stanford University, Stanford, CA). Mouse anti-TRAIL antibody used for Western blot was purchased from IMGENEX Corporation (San Diego, CA). Mouse antibodies against NFATc1, NFATc2 and NFATc4 were from Affinity BioReagents, Inc. (Golden, CO). Rabbit anti-β-actin antibody was from Sigma. Rabbit polyclonal anti-Sp1 antibody (07-645) was from Upstate (Lake Placid, NY). Mouse anti-NFATc3 antibody, the NFATc, NFATc mutant, Sp1, and ETS oligonucleotides were from Santa Cruz Biotechnology (Santa Cruz, CA). Human NFATc1, NFATc2, NFATc3, NFATc4, Sp1, and control siRNA were purchased from Dharmacon, Inc. (Lafayette, CO). [γ-^32^P] ATP (3,000 Ci/mmol) was from Amersham Pharmacia Biotech (Piscataway, NY). Immobilon-P nylon membranes for Western blots were purchased from Millipore (Bedford, MA), and x-ray film was purchased from Eastman Kodak (Rochester, NY). Total RNA was isolated using Ultraspec RNA from Biotecx Laboratories (Houston, TX). The enhanced chemiluminescence (ECL) system for Western immunoblot analysis was from Amersham (Arlington Heights, IL). Tissue culture media was obtained from GIBCO-BRL (Grand Island, NY). All other reagents were of molecular biology grade and purchased from either Sigma or Amresco (Solon, OH).

### Cell culture and treatment

Human colon cancer cell lines HT29 and Caco-2 (obtained from ATCC, Rockville, MD) were maintained in McCoy's 5A supplemented with 10% fetal calf serum (FCS) and MEM supplemented with 15% of FCS, respectively. The cells were pretreated with CsA for 30 min and then treated with PMA/Io in the presence or absence of CsA.

### Western blot analysis

Total protein was extracted and resolved on a 10% polyacrylamide gel and transferred to Immobilon-P nylon membranes as we have described previously [51]. Filters were incubated for 1 h at room temperature in blotting solution. TRAIL, Flag, Sp1, and β-actin were detected with specific antibodies following blotting with a horseradish peroxidase-conjugated secondary antibody and visualized using an ECL detection system.

### shRNAs and generation of stable cell lines

MISSION shRNA Lentiviral Particles with short-hairpin RNAs (shRNAs) were purchased from Sigma. The control shRNA (Non-Target shRNA Control Transduction Particles, catalog # SHC002V) contains 4 base pair mismatches within the short hairpin sequence to any known human or mouse genes. A set of four shRNAs to human NFATc1 (NM_006162; Sigma) were used; the sequences were as follows: CCGGCATCGAGATAACCTCGTGCTTCTCGAGAAGCACGAGGTTATCTCGATGTTTTT (TRCN0000017336), CCGGCGTCAGTTTCTACGTCTGCAACTCGAGTTGCAGACGTAGAAACTGACGTTTTT (TRCN0000017335), CCGGCCCGCCAACGTTCCAATTATACTCGAGTATAATTGGAACGTTGGCGGGTTTTT (TRCN0000017334), CCGGCGGCAACATTAGAAAGTGATTCTCGAGAATCACTTTCTAATGTTGCCGTTTTT (TRCN0000017333). HT29 cells were infected with the control shRNA or shRNA to human NFATc1 lentivirus particles and stably expressing cells were selected with puromycin at a concentration of 2 µg/ml.

### Preparation of nuclear extracts and electrophoretic mobility shift assays (EMSAs)

The nuclear extracts were prepared using a kit according to the manufacturer's protocol. EMSAs were performed as described previously [52] with minor modifications. Nuclear extracts (15 µg) were incubated with 40,000 cpm of ^32^P-labeled consensus oligonucleotide in a buffer containing 4% glycerol, 50 mM NaCl, 1 mM MgCl_2_, 0.5 mM dithiothreitol, 0.5 mM EDTA, 10 mM Tris-HCl; pH 7.5) and 0.5 µg/ml poly(dI·dC) in a final volume of 20 µl, for 15 min at room temperature. For supershift studies, 2 µl of antiserum was added to the nuclear protein for 20 min at room temperature prior to the addition of labeled probe. The complexes were fractionated on 6% native polyacrylamide gels run in 1× TBE buffer (89 mM Tris, 89 mM boric acid, and 2.0 mM EDTA), dried, and exposed to Kodak X-AR film at −70°C. Competition binding experiments were performed by the addition of the nonradioactive oligonucleotide, in 100-fold molar excess, at the time of addition of radioactive probe.

### Chromatin immunoprecipitation (ChIP) assay

The ChIP assay was performed using the ChIP-IT Express Enzymatic Kit (Active Motif) according to the manufacturer's protocol. PCR of the human TRAIL promoter containing the Sp1 binding sites was performed using total (input) or immunoprecipitated chromatin with the following pair of oligonucleotide primers: 5′- CATGGATCCTGAGGGCAAGG-3′ and 5′-TTGGCCCCACCCACATCTATTGAACC-3′.

### RNA isolation, real time PCR and reverse transcription-PCR (RT-PCR)

Total RNA was isolated using the Ultraspec™ RNA reagent. Real-time RT-PCR was performed using Applied Biosystems assays-on-demand 20× assay mix of primers and Taq-Man MGB probes (FAM™ dye-labeled) for target gene TRAIL (ID Hs00234355_m1) and predeveloped 18S rRNA(VIC™ dye-labeled probe) TaqMan® assay reagent (P/N 4319413E) for endogenous control. Separate tubes (singleplex) one-step RT-PCR was performed with 80 ng of RNA for both target gene and endogenous control. The reagent was TaqMan one-step RT-PCR master mix reagent kit (P/N 4309169). The cycling parameters for one-step RT-PCR were: reverse transcription 48°C for 30 min, AmpliTaq activation 95°C for 10 min, denaturation 95°C for 15 s, and annealing/extension 60°C for 1 min (repeat 40 times). Duplicate C_T_ values were analyzed in Microsoft Excel using the comparative C_T_(ΔΔC_T_) method as described by the manufacturer (Applied Biosystems). The amount of target (2^−ΔΔCT^) was obtained by normalizing to the endogenous reference (18S) and relative to a calibrator (one of the experimental samples).

RT-PCR was performed using a Titan One-Tube RT-PCR kit (Roche Applied Science, Indianapolis, IN) according to the manufacturer's protocol. Cycling conditions were as follows: 95°C for 30 s, 54°C for 30 s, and 68°C for 50 s for 31 cycles. Approximately 8 µl of product was run on a 0.5× Tris-borate-EDTA-1% agarose gel. Primer sets were synthesized by Sigma as follows: TRAIL (5′-CTTCACAGTGCTCCTGCAGT-3′ and 5′-TTAGCCAACTAAAAAGGCCCC-3′), NFATc1 (5′- GGAAGGGCGGCTTCTGCGAC-3′ and 5′- AGGCGTGCGGGCGCAGCAG-3′), NFATc2 (5′- CTGCCTCGCCACATCTACC-3′ and 5′-TGGTAGAAGGCGTGCGGCTT-3′), NFATc3 (5′-TGGATCTCAGTATCCTTTAA-3′ and 5′- TACACGAAACACAAGTCGTA-3′), NFATc4 (5′- TACAGATGTTCATCGGCAC-3′ and 5′ CGAAGCTCAATGTCTGAAT-3′).

Human β-actin was amplified to assess loading using the PCR primers supplied in the kit as follows: forward primer, 5′-CCAAGGCCAACCGCGAGAAGATGAC-3′; reverse primer, 5′-AGGGTACATGGTGGTGCCGCCAGAC.

### Plasmids, siRNAs, transfections, and dual-luciferase assay

The NFATc1, NFATc2, NFATc3, NFATc4 and control siRNA duplexes were introduced into HT29 cells by electroporation (Gene Pulser, Bio-Rad) as we have described previously [53]. TRAIL promoter reporter constructs and plasmids overexpressing NFATc1, NFATc2, NFATc3 and NFATc4 were transfected into cells using Lipofectamine 2000. The pRL-Tk-luc plasmid was co-transfected to normalize for variation in transfection efficiency. Luciferase assays were performed as described previously [54]. Briefly, 48 h after transfection, the cells were rinsed with PBS, harvested and lysed with 1× cell culture lysis reagent. Luciferase activity in 20 µl of extract was assayed with the dual luciferase assay system. Light emissions were integrated for the initial 10 s of emission using a Monolight 2010 luminometer (Analytical Luminescence Laboratory).

### Statistical analysis

Due to the heterogeneous variability among groups, data in Figure 3D and Figure 4D was analyzed using the Kruskal-Wallis test using SAS®, Release 9.1. Data presented in Figure 1A, 2B, 3F, 5E was analyzed using t test. Group differences were assessed at the 0.05 level of significance.
